# Architecture-Aware Augmentation: A Hybrid Deep Learning and Machine Learning Approach for Enhanced Parkinson’s Disease Detection

**DOI:** 10.3390/bioengineering11121218

**Published:** 2024-12-02

**Authors:** Madjda Khedimi, Tao Zhang, Hanine Merzougui, Xin Zhao, Yanzhang Geng, Khamsa Djaroudib, Pascal Lorenz

**Affiliations:** 1 Department of Electrical and Information Engineering, University of Tianjin, Tianjin 300072, China; zhangtao@tju.edu.cn (T.Z.); zhaoxin_16@tju.edu.cn (X.Z.); gregory@tju.edu.cn (Y.G.); 2 Department of Computer Science, University of Batna 2, Batna 05078, Algeria; h.merzougui@etu.univ-batna2.dz (H.M.); k.djaroudib@univ-batna2.dz (K.D.); 3 Department of Computer Science, IUT of Colmar University of Haute Alsace, 68008 Colmar, France; pascal.lorenz@uha.fr

**Keywords:** data augmentation, hybrid models, Parkinson’s disease, spiral drawings

## Abstract

Parkinson’s Disease (PD) is a progressive neurodegenerative disorder affecting millions worldwide. Early detection is crucial for improving patient outcomes. Spiral drawing analysis has emerged as a non-invasive tool to detect early motor impairments associated with PD. This study examines the performance of hybrid deep learning and machine learning models in detecting PD using spiral drawings, with a focus on the impact of data augmentation techniques. We compare the accuracy of Vision Transformer (ViT) with K-Nearest Neighbors (KNN), Convolutional Neural Networks (CNN) with Support Vector Machines (SVM), and Residual Neural Networks (ResNet-50) with Logistic Regression, evaluating their performance on both augmented and non-augmented data. Our findings reveal that ViT with KNN, initially achieving 96.77% accuracy on unaugmented data, experienced a notable decline across all augmentation techniques, suggesting it relies heavily on global patterns in spiral drawings. In contrast, ResNet-50 with Logistic Regression showed consistent improvement with data augmentation, reaching 93.55% accuracy when rotation and flipping techniques were applied. These results highlight that hybrid models respond differently to augmentation, and careful selection of augmentation strategies is necessary for optimizing model performance. Our study provides important insights into the development of reliable diagnostic tools for early PD detection, emphasizing the need for appropriate augmentation techniques in medical image analysis.

## 1. Introduction

Parkinson’s Disease (PD) is a progressive neurodegenerative disorder affecting over 10 million people worldwide, primarily impairing motor control due to the degeneration of dopaminergic neurons in the brain [1,2]. The early diagnosis of PD is crucial for improving patient outcomes, as treatments are currently limited to symptom management rather than a cure [3]. Among the earliest indicators of PD are fine motor impairments, such as micrographia and abnormalities in spiral drawing [4,5]. These motor dysfunctions can manifest years before the more widely recognized symptoms, such as tremors or bradykinesia, making spiral drawing analysis a promising non-invasive diagnostic tool [4]. Recent advancements in computer-aided diagnosis (CAD) systems have explored the potential of utilizing machine learning techniques to analyze spiral drawings [6,7]. Still, challenges remain in optimizing model performance and generalization, especially in the context of limited training data.

In the existing literature, traditional machine learning methods have been employed to extract handcrafted features from spiral drawings, focusing on measurable aspects such as stroke velocity, pressure, and curvature [8]. More recently, deep learning models, particularly Convolutional Neural Networks (CNNs), have gained traction due to their ability to automatically extract complex features and achieve higher diagnostic accuracy [9]. Despite their success, CNNs have limitations in capturing global patterns within spiral drawings, leading to the emergence of Vision Transformers (ViTs), which incorporate self-attention mechanisms to capture both local and global dependencies [10]. However, hybrid approaches that combine the strengths of both deep learning and traditional machine learning remain underexplored, especially concerning their response to data augmentation techniques—an essential strategy to address the limited availability of labeled medical data.

The current gap in the literature revolves around the impact of data augmentation on hybrid model performance in PD diagnosis using spiral drawing data. While data augmentation is widely recognized as a method for enhancing model generalization, its effects on different architectures, particularly hybrid models, are not well understood [11]. Some models, such as ViTs, may experience performance degradation when exposed to aggressive augmentations, while others, like CNNs, benefit from increased variability [12]. This study seeks to address this gap by systematically evaluating the performance of hybrid models under different data augmentation conditions, intending to identify optimal augmentation strategies for enhancing the diagnostic accuracy of spiral drawing-based PD detection tools.

The primary objective of this study is to investigate how different hybrid deep learning and machine learning models respond to various data augmentation techniques when applied to spiral drawing data for PD detection. Specifically, we aim to assess models such as ViT with K-Nearest Neighbors (KNN), CNN with Support Vector Machine (SVM), Residual Neural Network-50 (ResNet-50) with Logistic Regression, and Generative Adversarial Networks (GAN) with Random Forest, analyzing their performance with and without data augmentation. This research not only contributes to the growing body of work on hybrid machine learning architectures but also provides valuable insights into how augmentation strategies can be tailored to specific model architectures to improve diagnostic performance.

The rationale for this research lies in the potential for improved early diagnosis of PD through more accurate and generalizable machine learning models. Given the increasing interest in CAD systems for medical diagnosis, our study addresses a critical need to understand how model performance can be optimized through data augmentation, particularly in hybrid architectures that may offer better generalization in real-world applications. By advancing knowledge in this area, the research could lead to more reliable, accessible diagnostic tools, potentially transforming early detection practices for PD.

This paper is structured as follows: the Related Work section reviews relevant literature on spiral drawing as a diagnostic tool, machine learning models used in PD diagnosis, and the challenges of working with small datasets, including the role of data augmentation and learning rate schedulers. The Methodology section describes the dataset, the data augmentation techniques applied, the hybrid models used, and the evaluation metrics for assessing performance. The Discussion of Results section presents an analysis of the experiments, comparing the performance of models without data augmentation, with data augmentation, and an in-depth exploration of the impact of these techniques on different models. The Limitations section outlines the constraints faced in the study, followed by Future Work, which suggests avenues for further research. Finally, the Conclusion summarizes the key findings and contributions of the study.

This research aims to evaluate the impact of data augmentation on the performance of hybrid deep learning and machine learning models in diagnosing PD using spiral drawing data. By investigating the effectiveness of various augmentation techniques across different model architectures, this study seeks to identify the most suitable strategies for improving model generalization and diagnostic accuracy. Ultimately, the findings aim to advance the development of robust and reliable spiral drawing-based diagnostic tools, contributing to early detection efforts and enhancing clinical outcomes for PD patients.

## 2. The State of the Art

### 2.1. Spiral Drawing as a Diagnostic Tool

Spiral drawing tasks have become a cornerstone in the clinical assessment of PD detection [13]. Studies suggest that these tasks can effectively elicit the tremors and motor irregularities characteristic of PD [4]. The introduction of quantitative spiral analysis has allowed researchers to capture subtle motor impairments, such as reduced fluency and variability in pressure, that are often present before traditional clinical symptoms become apparent [5].

Toffoli et al. [5] introduce a novel approach utilizing a smart ink pen to capture more natural spiral drawings from patients. This technology, enriched with motion and force sensors, allows for more accurate measurements of motor function without altering the drawing task’s naturalness. The study found significant differences in the fluency and force applied by PD patients compared to healthy controls. Moreover, the classification models trained on these indicators achieved an accuracy of 94.38%, with fluency-related features emerging as key discriminators (Spiral drawing analysis).

Chakraborty et al. [14] also explored the use of spiral and wave drawings for PD diagnosis. The authors used convolutional neural networks (CNNs) to analyze the drawings, achieving a classification accuracy of 93.3% with an average F1-score of 93.94%. Their approach used a multistage classifier combining CNN and ensemble methods to improve detection performance. The results support the hypothesis that analyzing drawing patterns can serve as an effective non-invasive method for detecting PD in its early stages.

### 2.2. Machine Learning Models in PD Diagnosis

Several studies have applied machine learning (ML) techniques to analyze spiral drawings, with deep learning models showing promise in automating the diagnosis process. Huang et al. [15] evaluate the performance of six different deep learning models, including VGG16, VGG19, and ResNet architectures, using spiral drawing datasets. The study’s focus was on using data augmentation techniques like AugMix and PixMix to improve model generalization and accuracy. The VGG19 model achieved 89% accuracy with rotation and flipping augmentation applied to the spiral dataset.

Similarly, Farhah [16] explored the use of transfer learning models like VGG19, InceptionV3, ResNet50v2, and DenseNet169 in the classification of spiral drawings. Their study demonstrated that the InceptionV3 model achieved an accuracy of 89%, with an ROC curve value of 95%. Farhah’s work highlights the growing trend of using transfer learning techniques in medical image analysis, emphasizing the importance of pre-trained models in reducing training time and improving classification performance.

However, while the deep learning models demonstrated high classification accuracy, the study identified certain limitations. One key issue was the relatively small size of the dataset, which increased the risk of overfitting. Data augmentation was critical in addressing this limitation, but the researchers noted that larger datasets are needed for more robust results. Additionally, models like ResNet50 performed well, but their increased complexity made them more prone to overfitting when compared to shallower architectures like ResNet18.

### 2.3. Challenges with Small Datasets and Data Augmentation

One of the recurring challenges in spiral-based PD diagnosis research is the limited availability of large datasets. Many studies, including those reviewed here, rely on small datasets, which can hinder the generalizability of the results. Data augmentation techniques, such as rotation, flipping, and synthetic image generation, have been employed to address this issue.

Huang et al. [15] employed advanced augmentation techniques like AugMix, which improves model performance by creating more varied samples, thus enhancing the robustness and generalization of deep learning models. Despite this, models like Vit_base_patch16_224, which rely on ViTs, struggled with the small dataset, achieving lower accuracy (73.33%) compared to CNN-based models. This highlights the importance of both dataset size and augmentation strategy in developing effective diagnostic tools.

### 2.4. The Role of Data Augmentation and Learning Rate Schedulers

In their study, Huang et al. [15] also explored the role of learning rate schedulers, particularly cosine annealing, in improving model performance. Cosine annealing helps in preventing overfitting by gradually reducing the learning rate, allowing the model to converge more effectively on an optimal solution. This technique was found to be particularly effective when combined with data augmentation methods like AugMix, resulting in improved accuracy across most models, including VGG19, ResNet18, and ResNet50.

However, the study also notes that while these techniques improve performance on small datasets, they are no substitute for larger, more diverse datasets. The combination of augmentation techniques and learning rate schedulers can help mitigate some of the risks associated with small datasets but cannot fully compensate for the lack of data diversity.

A key gap identified in the literature is the limited size of datasets used in many of the studies. Small datasets not only increase the risk of overfitting but also limit the generalizability of findings to broader patient populations. Another limitation is the lack of comparative studies evaluating the performance of different deep-learning models on the same dataset. While some studies, such as Huang et al. [15], compare multiple models, there is a need for more systematic comparisons across a wider range of architecture and data acquisition techniques.

The following Table 1 provides a comparative overview of four key studies, illustrating the performance of different machine and deep learning models applied to spiral drawing datasets for PD diagnosis.

## 3. Materials and Methods

### 3.1. Methodology

**Figure 1:**
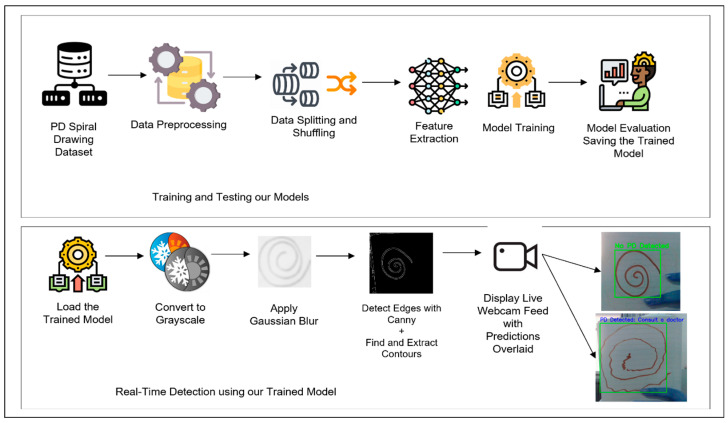
Our proposed work: overview of the complete system pipeline including training and real-time inference stages.

This study employs a hybrid approach that combines deep learning and machine learning techniques to improve the accuracy of PD detection through handwriting analysis, focusing on spiral drawings. Figure 1 outlines the complete workflow, from data preprocessing to real-time detection.

The dataset used in this study consists of spiral drawings labeled as either healthy (0) or PD (1). Preprocessing was conducted thoroughly to ensure uniformity and optimize the data for model training. Each image was labeled according to its respective category, and normalization was applied to scale pixel values to a range of [0, 1]. This step was essential for maintaining consistency across all models. The images were resized to 224 × 224 pixels to standardize input dimensions and meet the requirements of pretrained models such as ResNet-50. After resizing, the images were converted into tensor format for efficient processing within PyTorch. Additionally, the tensors were scaled to align with ImageNet standards by subtracting the dataset’s mean and dividing by its standard deviation for each color channel. This process ensures compatibility with pretrained models, improving their ability to work effectively with the dataset.

To address the limited dataset size, a detailed data augmentation strategy was applied to increase variability and reduce the risk of overfitting. Gaussian blur was introduced to mimic variations in drawing quality, while random rotations within a ±40-degree range accounted for differences in drawing orientations. Horizontal flipping with a 50% probability added diversity in spatial arrangement, and adjustments to brightness, contrast, and saturation simulated varying lighting conditions. These transformations were applied exclusively during the training phase to prevent any bias in the evaluation. Once preprocessing and augmentation were complete, the dataset was shuffled to eliminate ordering biases. It was then divided into training and testing subsets using a 70:30 ratio, ensuring enough data for effective model training while maintaining a sufficiently large test set for a reliable evaluation of generalization performance.

Several deep learning models were utilized for feature extraction. The Vision Transformer (ViT) divides input images into fixed-size patches and embeds each patch into a vector, which is processed through transformer layers to extract high-level features. The CLS token from the final hidden state summarizes the most important features from the entire image. Convolutional Neural Networks (CNNs) extract local features such as edges and textures, while deeper layers capture higher-level patterns like shapes and regions of interest. ResNet-50 builds on the CNN architecture with residual connections that allow the model to learn more complex features without encountering vanishing gradients. Generative Adversarial Networks (GANs) were also employed, where the generator creates synthetic spiral drawings and the discriminator refines these samples to closely resemble real data, thus enhancing dataset variability.

**Figure 2:**
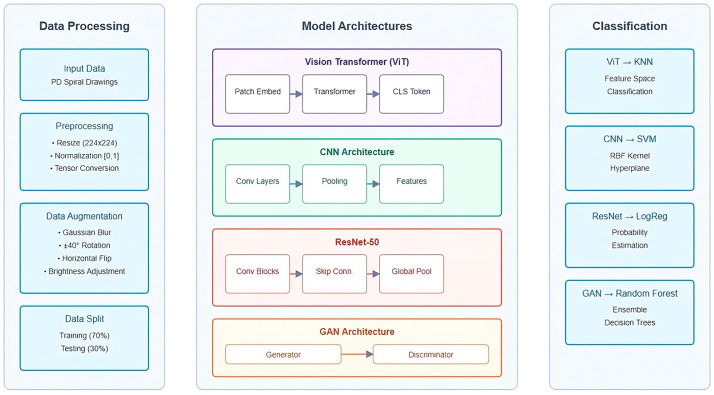
Our proposed work: overview of the complete system pipeline including training and real-time inference stages.

The complete architecture of our proposed PD spiral drawing detection system is illustrated in Figure 2, which outlines the data processing pipeline, the multiple deep learning models employed for feature extraction, and their corresponding classification approaches.

The extracted features were classified using traditional machine learning algorithms. ViT features were classified using K-Nearest Neighbors (KNN), which measures similarities in high-dimensional spaces. CNN features were paired with Support Vector Machines (SVMs) with a radial basis function kernel to handle non-linear data patterns. ResNet-50 features were classified using Logistic Regression for efficient binary classification. GAN-generated features were processed using Random Forest, a robust ensemble-based method well suited for complex datasets. The parameters of each classifier were fine-tuned using GridSearchCV to maximize performance.

### 3.2. Dataset

The dataset, curated by Adriano de Olivera Andrade and Joao Paulo Folado from the NIATS at the Federal University of Uberlândia, comprises spiral and wave drawings from individuals diagnosed with Parkinson’s Disease (PD) and healthy controls, and is sourced from Kaggle’s data repository. The dataset includes a total of 204 images, pre-split into a training set and a testing set, with each type of drawing (spiral and wave) containing 102 images. Specifically, each type is divided into 72 images for training and 30 images for testing.

For the purposes of this research, we focus exclusively on the spiral drawings, as they are widely recognized for their ability to capture key motor impairments associated with PD, such as tremors and irregular motor control.

**Figure 3:**
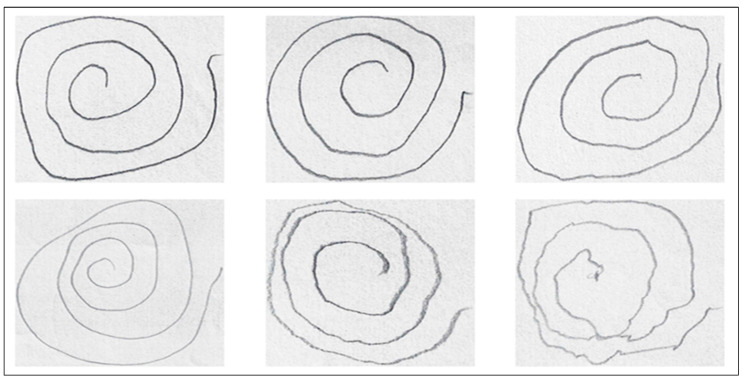
Hand-drawn spiral samples from individuals with and without PD.

Figure 3 illustrates variations in hand-drawn spiral patterns between individuals with PD and healthy controls, showcasing a sample from the dataset used.

**Figure 4:**
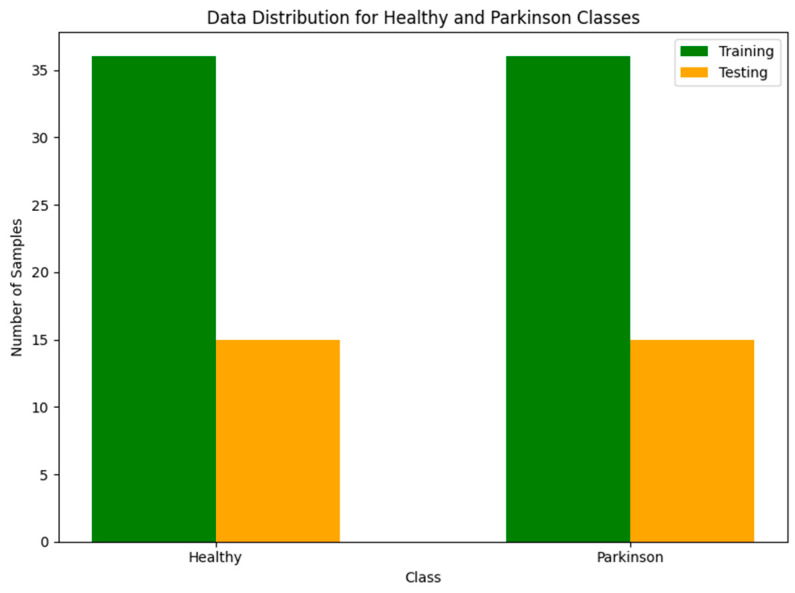
Distribution of healthy and Parkinson’s spiral drawings in the training and testing sets.

The dataset is balanced, with an equal number of images for each class in both the training and testing sets. Specifically, the training set contains 36 “healthy” and 36 “Parkinson’s” images, while the testing set includes 15 “healthy” and 15 “Parkinson’s” images. This balanced distribution ensures that the model is evaluated fairly without any class imbalance bias. A visual representation of the balanced dataset is shown in Figure 4.

The experiments were conducted on a personal laptop with an 11th Gen Intel(R) Core (TM) i7-1165G7 processor, 16 GB of RAM, and a 64-bit version of Windows 11 Pro.

For the software environment, Python 3.9 was used, along with libraries including TensorFlow for deep learning, Scikit-learn for machine learning tasks, and Matplotlib for data visualization. All experiments were carried out within Jupyter Notebook, providing an interactive environment for iterative model development and evaluation.

### 3.3. Data Augmentation

In the context of PD detection using spiral drawings, the challenge of limited datasets is significant [17,18,19]. This scarcity often leads to overfitting, where models perform poorly on unseen data [20]. To address this, we implemented a comprehensive data augmentation strategy to enhance the variability of the training set, thereby improving model robustness and generalizability.

Our augmentation pipeline was designed to simulate realistic variations in spiral drawing patterns that may arise from differences in orientation, lighting conditions, and individual writing styles. The process included several key transformations. All images were standardized to 224 × 224 pixels to ensure consistent input dimensions. A random horizontal flip, applied with 50% probability, introduced orientation variability. Images were rotated within a ±40-degree range to account for potential variations in positioning. Subtle modifications in brightness, contrast, and saturation were introduced to simulate diverse lighting conditions. Finally, images were normalized based on ImageNet dataset parameters, aligning with the pre-training standards of advanced models.

These augmentations were applied exclusively during the training phase to increase dataset diversity. During testing, only resizing and normalization were employed to ensure unbiased evaluation. By implementing this strategy, we aimed to address the limitations of small datasets, potentially enhancing the generalizability and clinical applicability of our PD detection models.

### 3.4. Hybrid Models

To enhance the diagnostic accuracy of PD detection from spiral drawings, we explored hybrid models that integrate deep learning-based feature extraction with traditional machine learning classifiers. The rationale for each combination lies in their complementary strengths, aiming to optimize both feature extraction and classification processes.

The ViT model paired with KNN classifier improves ViT’s ability to capture both local and global dependencies within images through its self-attention mechanism, making it effective for extracting nuanced features from complex spiral patterns. KNN was selected as the classifier due to its effectiveness in high-dimensional spaces, characteristic of ViT’s output feature vectors. This combination capitalizes on ViT’s advanced feature extraction while utilizing KNN’s adaptability in classification.

CNNs excel at extracting local features such as edges and textures, crucial for distinguishing between healthy and PD-affected spiral drawings. By pairing CNN with a SVM, particularly one using a radial basis function kernel, the model gains the ability to classify extracted features with high precision, leveraging SVM’s capacity to handle non-linear relationships in the data.

ResNet-50, with its deep residual learning framework, captures a wide range of feature hierarchies. These features are then fed into a Logistic Regression classifier, known for its efficiency in binary classification tasks. This combination offers both powerful feature extraction and computationally efficient classification.

GANs are employed to generate synthetic data that mimics the distribution of the real dataset, addressing the challenge of limited data availability. The features generated by the GAN are classified using Random Forest, an ensemble learning method known for its robustness with complex data. This approach aims to expand the feature space through GAN-generated data, allowing for potentially higher classification accuracy and stability.

By combining the strengths of deep learning and machine learning approaches, we aim to develop models that are not only accurate but also generalizable to a wider range of data scenarios in clinical settings.

### 3.5. Evaluation Metrics

To comprehensively assess the performance of the proposed hybrid models, we employed a diverse set of evaluation metrics. 

The confusion matrix is a fundamental tool in classification tasks, representing the relationship between the predicted and actual class labels [21]. For binary classification, the confusion matrix is mathematically expressed as follows:

(1) $$\left[\begin{array}{cc} TP & FN\\ FP & TN \end{array}\right]$$

where:

- TP (True Positives) refers to the number of instances correctly predicted as positive.
- FN (False Negatives) denotes the number of positive instances incorrectly classified as negative.
- FP (False Positives) represents the number of negative instances incorrectly predicted as positive.
- TN (True Negatives) indicates the number of instances correctly predicted as negative.

Accuracy is a fundamental metric that reflects the overall proportion of correct predictions made by the model. It is calculated as the ratio of correctly predicted instances (both true positives and true negatives) to the total number of instances. Although accuracy is widely used, it may provide a misleading assessment of model performance, particularly in imbalanced datasets, as it does not account for the distribution of false positives and false negatives [22,23]. The formula for accuracy is:

(2) $$Accuracy=\frac{Number\;of\;Correct\;Predictions}{Total\;Number\;of\;Predictions}$$

Recall (Sensitivity or True Positive Rate) measures the model’s ability to correctly identify positive instances. In medical diagnostics, high recall is crucial, as it minimizes the risk of false negatives, which could delay diagnosis or treatment, potentially worsening patient outcomes [24]. Recall is calculated as the ratio of true positives to the sum of true positives and false negatives:

(3) $$Recall\;(TPR)=\frac{TP}{TP+FN}$$

Precision quantifies the accuracy of positive predictions made by the model. It measures the proportion of predicted positive instances that are truly positive. High precision is particularly important in medical contexts to avoid unnecessary treatment or interventions caused by false positives [24]. It is calculated as the ratio of true positives to the sum of true positives and false positives:

(4) $$Precision=\frac{TP}{TP+FP}$$

The F1-Score is the harmonic mean of precision and recall, providing a balanced evaluation of the model’s performance, especially in situations where the classes are imbalanced. It combines both false positives and false negatives into a single metric, making it particularly useful when there is a need to balance precision and recall [25]. The F1-Score is calculated as:

(5) $$F1\;Score=2\times \frac{Precision\times Recall}{Precision+Recall}$$

Finally, the Area Under the Curve-Receiver Operating Characteristic (AUC-ROC) quantifies the overall performance of a binary classification model in distinguishing between positive and negative classes [26,27]. It is computed as the area under the ROC curve, which plots the True Positive Rate (TPR) against the False Positive Rate (FPR) at various decision thresholds. The mathematical representation of the AUC is:

(6) $$AUC=\int_{0}^{1}TPR(FPR)d(FPR)$$

where:

- FPR (False Positive Rate) is defined as:

(7) $$FPR=\frac{FP}{FP+TN}$$

In this study, we prioritize reporting Precision, Recall, F1-Score and AUC-ROC over training accuracy. Even with a balanced dataset, these metrics provide a more comprehensive evaluation of model performance. They offer insights into the model’s ability to correctly identify positive and negative cases (Precision and Recall), balance between these measures (F1-Score) and distinguish between classes (AUC-ROC). While training accuracy can be informative, it may not always reflect the model’s true predictive power on unseen data. Our chosen metrics ensure a thorough assessment of the model’s real-world applicability and generalization capabilities.

## 4. Discussion

This section analyzes the experimental results obtained from the hybrid deep learning and machine learning models for detecting PD using spiral drawing samples. We explored two distinct scenarios: one where no data augmentation was applied and another where data augmentation techniques were utilized to artificially increase the variability of the training data. The aim is to understand how different model architectures respond to the variability in data and to identify patterns in their performance across several key metrics, including accuracy, precision, recall, F1-score and AUC-ROC.

### 4.1. Experiments Without Data Augmentation

This section analyzes the performance of hybrid models for PD detection from spiral drawings, comparing results with and without data augmentation.

The initial experiments evaluated the hybrid models using the unaugmented dataset, providing insight into their ability to learn from the inherent variability of the original data. The performance metrics for each model are summarized in Table 2.

In our initial experiments, we evaluated the performance of our hybrid models using the dataset without any data augmentation. This approach allowed us to assess each model’s ability to detect PD from spiral drawings using only the inherent variability present in the original data.

The results revealed a clear distinction in performance among the different hybrid combinations. The ViT with KNN emerged as the top performer, closely followed by ResNet-50 with Logistic Regression. Both models achieved impressive levels of accuracy (96.77%) and precision (100%) for Class 0, demonstrating a robust capacity to identify PD cases without the need for artificially enhanced data variability. ViT with KNN achieved a perfect precision (100%) and recall (100%) for Class 1 as well, indicating it was equally effective in identifying non-PD cases.

Notably, ViT with KNN showed a slight edge in discriminative power, as evidenced by its higher AUC-ROC score of 99.58% compared to ResNet-50 with Logistic Regression (98.74). This suggests that ViT with KNN may have a marginal advantage in distinguishing between PD and non-PD cases across various classification thresholds. The F1-scores for Class 0 and Class 1 were 97% for both classes, reflecting the model’s balanced performance in both categories.

In contrast, CNN with SVM and GAN with Random Forest showed comparatively lower accuracy, 87.10% and 83.87%, respectively. These models demonstrated significant performance gaps, particularly in terms of precision and recall for Class 1. For CNN with SVM, the precision for Class 0 was 93%, but the recall for Class 1 was 93%, indicating good performance in identifying non-PD cases but relatively lower ability to detect PD cases. Similarly, GAN with Random Forest struggled with lower recall for Class 0 (76%), suggesting that it missed a considerable portion of PD cases, although it performed better for Class 1 with a recall of 93%.

The F1-scores for CNN with SVM and GAN with Random Forest were 0.88 and 0.84, respectively. These results point to areas where further refinement could yield significant improvements, particularly in detecting Class 0 cases more accurately. For CNN with SVM, the lower F1-score for Class 1 suggests that it might benefit from a more balanced approach to detecting both classes, while GAN with Random Forest could likely benefit from more diverse data to improve its classification performance.

These findings highlight the importance of model architecture in handling unaugmented data for PD detection. The superior performance of ViT with KNN, particularly its higher AUC-ROC score, indicates its potential as a highly reliable tool for PD diagnosis, even in scenarios where data augmentation might not be feasible. The strong performance of ResNet-50 with Logistic Regression also suggests its viability as a diagnostic tool. Conversely, the results for CNN with SVM and GAN with Random Forest, with their lower recall for Class 0 and lower F1-scores for both classes, point to areas where further refinement could improve diagnostic accuracy, especially in identifying PD cases (Class 0).

### 4.2. Experiments with Data Augmentation

In our subsequent experiments, we applied various data augmentation techniques during training to simulate real-world variations in spiral drawings. The results in Table 3 revealed interesting patterns in how different hybrid models respond to various augmentation methods.

#### 4.2.1. ViT with KNN

Initially the best-performing model, ViT-KNN experienced consistent performance degradation after augmentation. Accuracy dropped from 96.77% pre-augmentation to 77.42% (AugMix) and 87.10% (CutMix). Despite reduced accuracy, precision, recall, and F1-scores for Class 0 and Class 1 remained balanced under CutMix (e.g., F1-scores of 88% for both classes). Its AUC-ROC values were still strong, reaching 95.38% (CutMix) and 90.13% (AugMix). This highlights the model’s ability to maintain classification robustness despite augmentation challenges.

#### 4.2.2. CNN with SVM

The CNN-SVM model showed variation in response to augmentation. Rotation and Flipping enhanced accuracy to 90.32%, with precision reaching 94% (Class 0) and recall for Class 1 at 93%. F1-scores remained balanced at 91% (Class 0) and 90% (Class 1). Under Gaussian Blur, it maintained a baseline accuracy of 87.10%, but precision for Class 0 improved to 100%, with corresponding F1-scores of 87% for both classes. Conversely, CutMix lowered accuracy to 80.65%, accompanied by reduced recall and F1-scores. Despite these inconsistencies, the AUC-ROC values for Gaussian Blur (96.22%) and Rotation and Flipping (94.96%) were robust.

#### 4.2.3. ResNet-50 with Logistic Regression

This combination demonstrated consistent improvement across most augmentation techniques. With Rotation and Flipping, it achieved the highest accuracy of 93.55%, perfect precision for Class 0 (100%), and strong recall for both classes (88% for Class 0, 100% for Class 1). The F1-scores for Class 0 and Class 1 were 94% and 93%, respectively, with an AUC-ROC of 97.48%. Under Gaussian Blur and AugMix, the model maintained solid performance, with accuracies of 90.32%, high AUC-ROC values (98.32% and 95.38%), and balanced metrics. However, CutMix caused a slight degradation, lowering accuracy to 80.65% and F1-scores to 82% (Class 0) and 79% (Class 1).

#### 4.2.4. GAN with Random Forest

The GAN-Random Forest hybrid showed limited benefit from augmentation. It consistently achieved an accuracy of 87.10% under Rotation and Flipping, Gaussian Blur, and AugMix, maintaining precision and recall values of 93% (Class 0) and 81% (Class 1). F1-scores for both classes remained steady at 88% and 87%, respectively, with high AUC-ROC scores, peaking at 96.64% (Gaussian Blur). However, CutMix significantly degraded performance, with accuracy dropping to 67.74%, reflecting reductions in recall and F1-scores.

**Figure 5:**
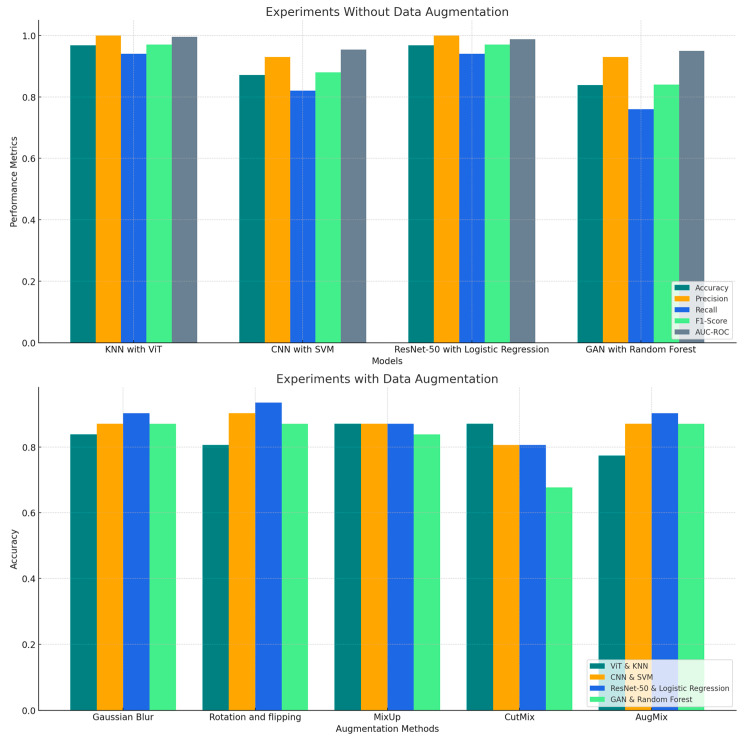
Comparative performance of hybrid models in PD detection with and without data augmentation techniques.

The comparative analysis in Figure 5 highlights the variability in model performance across different evaluation metrics with and without data augmentation. 

ResNet-50 with Logistic Regression consistently outperforms other models, especially when augmented with techniques like Gaussian Blur and Rotation & Flipping. Among the augmentation techniques, Rotation & Flipping proved to be the most consistently beneficial, particularly for CNN with SVM and ResNet-50 with Logistic Regression. AugMix and Gaussian Blur also had positive effects on most models, except for ViT with KNN, which exhibited reduced performance variability, indicating its sensitivity to augmentation strategies. 

**Figure 6:**
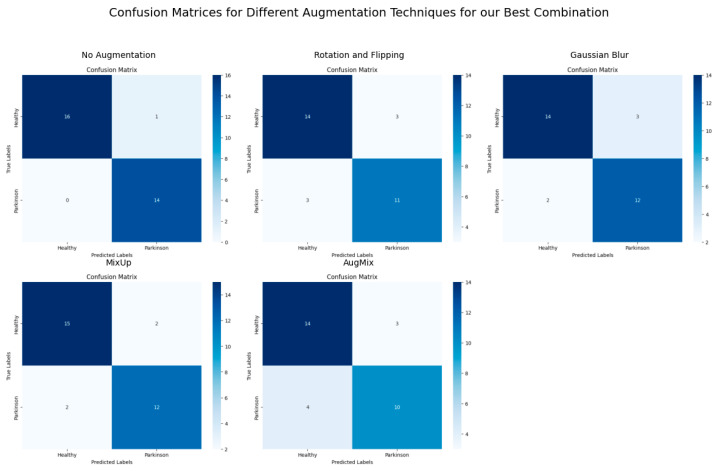
Confusion matrices for ViT with KNN across different augmentation techniques.

**Figure 7:**
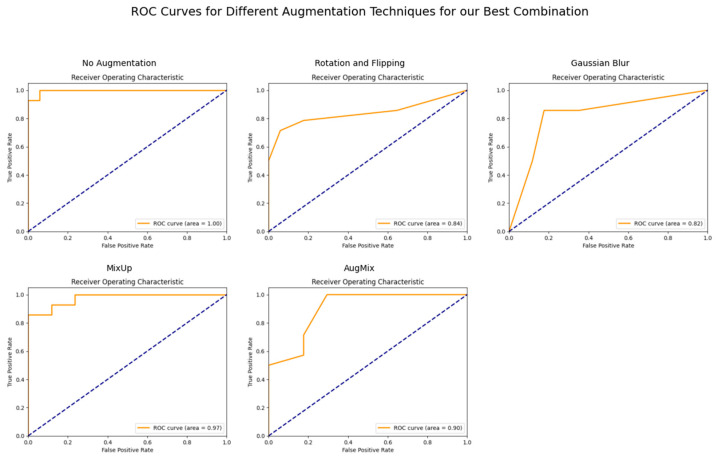
ROC Curves for ViT with KNN across Different Augmentation Techniques.

**Figure 8:**
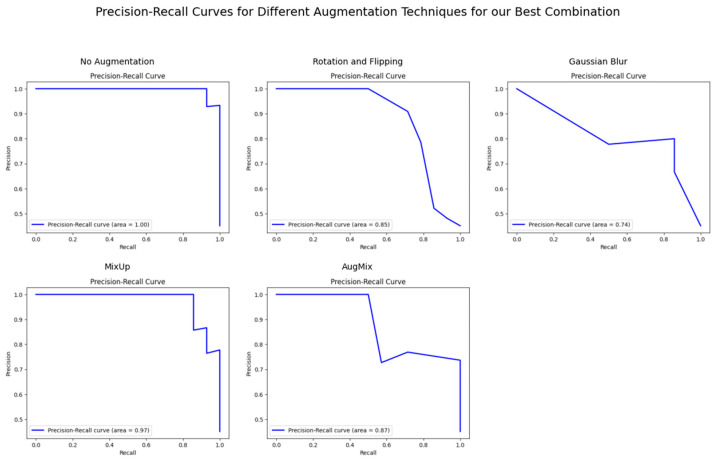
Precision-recall curves for ViT with KNN across different augmentation techniques.

In contrast, CutMix had more varied impacts, with CutMix notably reducing performance for GAN with Random Forest. Figure 6, Figure 7 and Figure 8 demonstrate the ViT-KNN model’s performance through confusion matrices, ROC curves, and precision-recall curves, providing a multi-metric evaluation of our best-performing combination.

Figure 6 shows the classification effectiveness of ViT with KNN for each augmentation method. The Confusion Matrix for the non-augmented dataset highlights near-perfect classification, with 16 healthy subjects and 14 Parkinson’s subjects correctly classified. With augmentation techniques like CutMix, the model still maintains a high level of classification accuracy, with only a few misclassifications compared to more challenging techniques like AugMix, where more errors are observed.

Figure 7 illustrates the Receiver Operating Characteristic curves for the different augmentation methods. The ROC curve for the non-augmented data demonstrates perfect classification with an AUC of 1.00. Similarly, CutMix shows excellent ROC AUC scores of 0.97 and 0.95, with the model maintaining a high true positive rate and low false positive rate. AugMix, on the other hand, causes a noticeable drop in performance, reflected in the lower ROC AUC of 0.77.

Figure 8 complements the ROC analysis by focusing on the model’s precision and recall performance. In the non-augmented dataset, the Precision-Recall curve reveals a perfect balance with an AUC of 1.00. CutMix maintains strong performance, with AUC scores of 0.97 and 0.92, indicating a solid balance between precision and recall. Nevertheless, in the case of AugMix, the performance declines, showcasing the model’s sensitivity to this specific augmentation method.

### 4.3. Understanding the Impact of Data Augmentation on Different Models

The performance of hybrid models under augmented and non-augmented conditions provides valuable insights into the interaction between model architecture and data augmentation techniques in the context of PD detection from spiral drawings.

The ViT with KNN exhibited a notable decline in performance across all augmentation methods. This unexpected behavior proves the importance of preserving global spatial structures in spiral drawings for this model. ViT’s reliance on capturing global patterns appears to be disrupted by common augmentation techniques, suggesting that for models heavily dependent on the global context, augmentation strategies must be carefully tailored to avoid distorting critical diagnostic features.

In contrast, the ResNet-50 combined with Logistic Regression demonstrated remarkable consistency in performance across various augmentation techniques. This combination maintained high accuracy levels, with notable improvements when using rotation and flipping (93.55%) and consistent performance with Gaussian blur and AugMix (90.32%). The robust performance of this hybrid model, both with and without augmentation, highlights its adaptability and reliability for PD detection. The deep feature extraction capabilities of ResNet-50, coupled with the straightforward classification approach of Logistic Regression, appear to create a synergy that effectively handles various data representations.

The CNN with SVM combination showed mixed results with data augmentation. While it achieved its highest accuracy (90.32%) with rotation and flipping, surpassing its baseline performance, other augmentation techniques yielded results comparable to or slightly below its non-augmented performance. This varied response suggests that CNN’s effectiveness is closely tied to the specific type of local feature variability introduced by different augmentation methods. The improvement observed with certain augmentations indicates that exposing the model to a broader range of spiral drawing variations can enhance its ability to learn generalizable features, potentially leading to improved classification performance in some cases.

The GAN paired with Random Forest demonstrated modest gains with certain augmentation techniques, matching its baseline performance (87.10%) with rotation and flipping, Gaussian blur, and AugMix. However, it struggled significantly with CutMix, indicating a sensitivity to particular types of data manipulation. These results suggest that while GANs can generate diverse synthetic features, the effectiveness of this approach is closely tied to the quality and nature of the input data and the specific augmentation method used.

These findings highlight the complex relationship between model architecture and data augmentation in the context of PD detection from spiral drawings. They emphasize the need for careful consideration when selecting augmentation strategies, as their impact can vary significantly depending on the underlying model architecture.

### 4.4. Complexity Analysis of Hybrid Models and Data Augmentation Techniques

The execution times highlight the varying complexities of the hybrid models and the impact of data augmentation techniques.

ViT with KNN demonstrates moderate execution times (7.82–8.82 s), balancing the computational demands of ViT’s attention mechanisms and KNN’s simplicity. CNN with SVM is the most efficient (2.15–3.09 s), owing to CNN’s lightweight convolutional operations and SVM’s straightforward classification.

ResNet-50 with Logistic Regression performs efficiently with augmentations (2.90–3.59 s) but displays an anomaly without augmentation (2618.01 s), indicating potential inefficiencies in baseline processing. GAN with Random Forest is the most computationally intensive (901.56–3382.87 s), driven by GAN’s iterative adversarial training and Random Forest’s complexity.

Augmentations such as CutMix and AugMix add minimal overhead while improving input diversity. However, their impact is more significant in computationally heavy models. The choice of a hybrid model depends on the trade-off between execution speed and the need for robust feature extraction and classification.

## 5. Conclusions

The results of this study reveal that data augmentation has a variable impact on different hybrid models, depending on their architecture and feature extraction processes. ViT with KNN performed better without data augmentation due to its reliance on global spatial structures, which are disrupted by aggressive augmentation techniques. On the other hand, models like ResNet-50 with Logistic Regression and CNN with SVM demonstrated significant performance gains with data augmentation, highlighting their ability to leverage increased data variability for improved generalization. GAN with Random Forest benefited modestly from augmentation, suggesting that while the increased feature diversity was helpful, the model may still be constrained by the quality of the synthetic data generated by the GAN. 

This study has multiple limitations that may impact on the interpretation and generalizability of our findings. The first concern is the limited amount of data available for training and testing (the dataset is small), which contributes to overfitting in training and constraining generalization abilities to new datasets. The dataset may not be entirely representative of the varied population of patients with PD, and differences in demographics and disease phenotype can affect model performance. The complexity of the models, especially with architectures such as ViTs, can also see poor performance under heavy data augmentation. Additionally, as accuracy does not tell the full story, the availability of uniform reporting of other metrics like precision and recall is limited, making meta-comparisons nearly incomplete. Finally, the lack of external validation means that it is unclear whether these findings can be replicated in other clinical settings. 

The complexity analysis reveals substantial differences in computational efficiency across hybrid models, with simpler architectures like CNN with SVM and ViT with KNN demonstrating faster and more efficient execution, while computationally intensive models such as GAN with Random Forest require significantly more resources. These findings emphasize the importance of matching model complexity to specific task requirements for optimal performance.

There are several important areas for future research building on these findings. For instance, the model generalization and reliability could be improved by collecting larger as well as more diverse datasets. Comparative studies on machine learning architectures systematically designed to study their ability to generalize over different datasets in an identical environment are necessary to better understand the architecture scale. Further investigating more architecture and innovative data augmentation methods (like GANs) for designing models that are less sensitive to noise could be beneficial as well. Combining multiple types of data, including clinical assessments and neuroimaging, could also help improve diagnostic accuracy. Lastly, longitudinal studies designed to follow spiral drawing change over time could provide important information on the progression of disease and aid in further developing predictive models. Targeting these regions will lead to more precise PD diagnostic tools, hence better results for patients as well.

## Figures and Tables

**Table 1:** Comparative overview of state-of-the-art models for PD diagnosis using spiral drawing datasets.

Papers with APA Citation	Dataset	Best Model	Accuracy (%)	Precision (%)	Recall (%)	F1 Score (%)
Huang et al. [15]	The NIATS dataset (Same dataset)	VGG19	87.66	92	80	N/A
Farhah, N. [16]	The NIATS dataset (Same dataset)	InceptionV3	89	78	100	88
Toffoli et al. [5]	Custom dataset created by the authors	Catboost with the SHAP (Shapley Additive Explanation) technique	94.38	90.63	100	95.08
Chakraborty et al. [14]	The NIATS dataset (Same dataset)	CNN with Meta Classifier (Voting Ensemble)	93.3	93.5	94	93.94

**Table 2:** Performance metrics of hybrid models for PD detection from spiral drawings before data augmentation.

Hybrid Model	Accuracy (%)	Precision (Class 0) (%)	Precision (Class 1) (%)	Recall (Class 0) (%)	Recall (Class 1) (%)	F1-Score (Class 0) (%)	F1-Score (Class 1) (%)	AUC-ROC (%)
ViT with KNN	96.77	100	93	94	100	97	97	99.58
CNN with SVM	87.10	93	81	82	93	88	87	95.38
ResNet-50 with Logistic Regression	96.77	100	93	94	100	97	97	98.74
GAN with Random Forest	83.87	93	76	76	93	84	84	94.96

**Table 3:** Performance metrics of hybrid models for PD detection from spiral drawings after data augmentation.

Hybrid Model	Augmentation	Accuracy (%)	Precision (Class 0) (%)	Precision (Class 1) (%)	Recall (Class 0) (%)	Recall (Class 1) (%)	F1-Score (Class 0) (%)	F1-Score (Class 1) (%)	AUC-ROC (%)
ViT with KNN	Rotation and Flipping	80.65	82	79	82	79	82	79	83.82
	AugMix	77.42	78	77	82	71	80	74	90.13
	CutMix	87.10	88	86	88	86	88	86	95.38
	Gaussian Blur	83.87	88	80	82	86	85	83	82.14
CNN with SVM	Rotation and Flipping	90.32	94	87	88	93	91	90	94.96
	AugMix	87.10	93	81	82	93	88	87	93.70
	CutMix	80.65	88	73	78	85	82	79	84.19
	Gaussian Blur	87.10	100	78	76	100	87	88	96.22
ResNet-50 with Logistic Regression	Rotation and Flipping	93.55	100	88	88	100	94	93	97.48
	AugMix	90.32	100	82	82	100	90	90	95.38
	CutMix	80.65	100	65	70	100	82	79	87.73
	Gaussian Blur	90.32	94	87	88	93	91	90	98.32
GAN with Random Forest	Rotation and Flipping	87.10	93	81	82	93	88	87	92.44
	AugMix	87.10	93	81	82	93	88	87	92.86
	CutMix	87.10	92	83	80	94	86	88	95.83
	Gaussian Blur	87.10	100	78	76	100	87	88	96.64

## Data Availability

The data presented in this study are available at https://www.kaggle.com/code/basel99/parkinson-s-disease-detection/input (accessed on 20 August 2024).
